# The Importance of Self-Monitoring for Behavior Change in Youth: Findings from the SWITCH^®^ School Wellness Feasibility Study

**DOI:** 10.3390/ijerph16203806

**Published:** 2019-10-10

**Authors:** Gabriella M. McLoughlin, Richard R. Rosenkranz, Joey A. Lee, Maren M. Wolff, Senlin Chen, David A. Dzewaltowski, Spyridoula Vazou, Lorraine Lanningham-Foster, Douglas A. Gentile, Marisa S. Rosen, Gregory J. Welk

**Affiliations:** 1 Department of Kinesiology, Iowa State University, Ames, IA 50011, USA; svazou@iastate.edu (S.V.); gwelk@iastate.edu (G.J.W.); 2 Department of Food, Nutrition, Dietetics & Health, Kansas State University, Manhattan, KS 66506, USA; ricardo@ksu.edu; 3 Helen & Arthur E. Johnson Beth-El College of Nursing and Health Sciences, University of Colorado, Colorado Springs, CO 80918, USA; jlee29@uccs.edu; 4 Department of Food Science and Human Nutrition, Iowa State University, Ames, IA 50011, USA; mmwolff@iastate.edu (M.M.W.); lmlf@iastate.edu (L.L.-F.); 5 School of Kinesiology, Louisiana State University, Baton Rouge, LA 70803, USA; senlinchen@lsu.edu; 6 University of Nebraska Medical Center, Omaha, NE 68198-4365, USA; david.dzewaltowski@unmc.edu (D.A.D.); marisa@familyplanningcouncilne.org (M.S.R.); 7 Department of Psychology, Iowa State University, Ames, IA 50011, USA; dgentile@iastate.edu

**Keywords:** dissemination and implementation, health promotion, obesity prevention, physical activity, school wellness, sedentary behavior

## Abstract

School Wellness Integration Targeting Child Health (SWITCH^®^) is a school wellness implementation initiative focused on building capacity for schools to plan and coordinate wellness programming. Grounded in Social Cognitive Theory (SCT), the purpose of this study was to evaluate the utility of the web-based, self-regulation system on physical activity (PA) behavior outcomes. At pre-test and post-test, students in SWITCH^®^ schools (*n* = 8) completed the online Youth Activity Profile (YAP) to assess PA and sedentary behavior (SB). Students (*n* = 513) were categorized into high or low self-monitoring groups (using a median split) based on their use of the web-based self-regulation platform. Linear mixed models were used to assess differences in moderate-to-vigorous PA (MVPA) and sedentary behavior, with school, classroom, student, time-by-school, and time-by-classroom random effects and main and interaction fixed effects for student self-monitoring, gender, and time. Significant self-monitoring-by-time interactions were observed for estimates of PA F(1, 477) = 5.55, *p* = 0.02 and SB F(1, 477) = 4.90, *p* = 0.03. Students in the high self-monitoring group had larger gains in PA per day and larger declines in hours per day of sedentary screen time behavior compared to students in the low self-monitoring group. These findings support the utility of web-based self-regulation for facilitating PA change in youth.

## 1. Introduction

There is a clear need to develop effective and feasible strategies to promote healthy lifestyles in children [1]. Schools provide an ideal setting for coordinated youth obesity prevention, but it has proven difficult to disseminate evidence-based programs widely in a cost-effective way [2,3]. Current recommendations emphasize the advantages of whole-of-school approaches [4,5] as opposed to isolated interventions in specific settings such as physical education, before- and after-school programs, or recess. The advantage of whole-of-school approaches is that they focus on engaging teachers, administrators, other staff (e.g., school nurses, food service managers/directors), students, and parents to provide a health-promoting environment for students; such approaches also integrate an array of resources and programming to help build the capacity of students to perform healthy behaviors. Whole-of-school interventions that target multiple behaviors and reach multiple settings offer the most promise for larger and more sustained impact; however, research suggest that schools need training and support to be able to plan and support wellness programming [6,7].

A promising whole-of-school intervention with potential for dissemination is the “Switch” obesity prevention program [8]. Consistent with social–ecological models, “Switch” was designed to reach multiple settings within schools while also facilitating engagement with families and community partners. The program focuses on three distinct behaviors known to have an impact on obesity (i.e., physical activity (PA), sedentary behavior (SB), and fruit and vegetable consumption) in a creative way by challenging children to “switch what they do, view and chew” [8]. In a cluster-randomized controlled trial, youth exposed to the “Switch” program had significantly more favorable outcomes in both SB (screen time) and fruits/vegetable consumption than students in control schools—and these effects were sustained for six months following the intervention [9].

A limitation of the original “Switch” program was that the print-based materials made it cost-prohibitive to promote broader dissemination. Therefore, subsequent work has been focused on transitioning the intervention to an online package that could be more widely disseminated. In a controlled effectiveness study, we demonstrated that a web-based version of “Switch” had similar utility and outcomes as the print-based program; however, effects were directly related to the degree of engagement within the school [10]. Through a United States Department of Agriculture (USDA) funded project, we developed a novel training model designed to facilitate greater adoption and more effective implementation in schools. The model was based on the Healthy Youth Places Model, a prior school-based capacity-building model for implementation of a health promotion system and practice changes [11,12]. Accordingly, the focus in this revised conception of School Wellness Integration Targeting Child Health (SWITCH^®^) is on building capacity for schools to plan and coordinate wellness programming on their own [13].

The training model comprises of a series of online webinars and in-person training to facilitate capacity building in school wellness teams [13]. School wellness teams undergo school wellness training in the fall of each academic year, followed by implementation in the following spring. Training webinars provide wellness teams with content knowledge on the three themes of “do, view, and chew” and how to promote these concepts through educational modules for the classroom, physical education, and lunchroom settings, respectively. In-person training takes place at a conference on the Iowa State campus where wellness teams are educated on the quality elements and best practices. Quality elements are derived from factors that positively influence student health outcomes, such as: regular team meetings, use of promotional materials such as posters and trinkets, facilitating integration of SWITCH^®^ across the school environment, and engaging parents and students as key stakeholders [10,13]. In a feasibility study, our team found that schools found this training method beneficial and enabled them to implement SWITCH^®^ with latitude in ways that complemented existing programs and practices [13]. For instance, schools could adopt/implement as many lessons they felt comfortable with and in any or all of the three educational settings (classroom, PE, lunchroom). They were also able to distribute trinkets and use posters in whatever way suited their existing school structure and schedule. Finally, involvement of students and parents was discretionary, but schools were provided with training on ways in which they could engage these stakeholders. Such flexibility enhanced school wellness team’s perceptions of the program and perceived competence to continue wellness programming on their own [13].

In the 2017 iteration of SWITCH^®^, a hybrid type II design (one of the three hybrid effectiveness and implementation designs [14]), was used to evaluate the feasibility of the distributed training model, while also testing other assumptions in our approach. The focus in this paper was on the utility of the web-based self-regulation platform for facilitating student-level behavior change. Experts in intervention research have specifically emphasized the need to evaluate the intervention components prior to dissemination of evidence-based programs [15]. The original “Switch” project utilized printed behavioral trackers to facilitate children’s self-monitoring and goal setting, but these features have now been embedded within the web-based customized SWITCH^®^ online system to facilitate behavior change. Further, although self-regulation (including self-monitoring, self-comparison of monitoring a result to a goal, and self-judgement) have an established evidence base within a range of populations for a broad array of targeted health-promoting behaviors [16], it was important to directly evaluate the utility of this new web-based platform for promoting behavior change within the school setting. Evaluation of how schools and teachers facilitate self-regulation of students is also imperative to assess the ecological validity of online self-monitoring as a means to enhance health behavior and to take evidence-based programs to scale.

The present study tests the utility of the SWITCH^®^ web-based self-regulation system for promoting PA and SB change in youth within the context of a whole-of-school wellness implementation framework. The study is grounded in Social Cognitive Theory (SCT [17]) which includes self-regulation as a relevant process for promoting health behavior change [18]. SCT can be useful to frame interventions to change health behaviors based on the tenet of triadic reciprocal determinism among people, their behaviors, and their environments [17]. Core theoretical constructs of SCT include individual outcome expectations of making changes to health behavior (what people expect to result from behavior change), self-efficacy to make health behavior change (how confident people are in changing behavior, particularly when faced with various challenges), and behavioral capability (possessing the skills and knowledge to perform a behavior). These behavioral determinants are frequently accompanied by various facilitators and barriers that may help or hinder the achievement of positive health behaviors [17].

As a follow-up to the original conception of SCT, Bandura outlined multiple applications of SCT to promote health behaviors in various populations [18]; one application addresses the concept of self-management, whereby people monitor and get feedback on heath behaviors, then set goals, and learn to manage and improve their health and wellbeing. Researchers have conducted self-management and goal setting interventions in clinical settings with adults through web-based platforms [19,20] but a paucity of such work has been documented among children. The SWITCH^®^ monitoring system provides an ideal way to evaluate this mechanism since students monitor their behavior, set goals, and receive feedback for self-judgment through the integrated system. Through the web interface, children see summaries of the days in which they met the SWITCH^®^ goal and days they did not. They can also set behavioral ‘switches’ that help them plan ways to be more active that week. Examples of switches include cooking a healthy meal together with family (chew/view) and walking to school one day each week (do). Monitoring is reinforced at the class level by providing class ‘badges’ based on percentages of youth that track and that record ‘switches’. This provides a way for teachers to prompt and reinforce both individual and class-level engagement in self-monitoring. During the training phase of SWITCH^®^, teachers are shown how to view their students’ activity and are able to see which students have engaged in self-monitoring behavior. They may reinforce these monitoring efforts, but this is at their own discretion. While previous research has used web-based platforms to promote self-regulation in adults [19,20], it has not been directly evaluated in children. Thus, the purpose of this study was to elucidate the relationship between self-regulation techniques though online behavioral self-monitoring and feedback (within school classrooms) and changes in children’s PA and SB.

## 2. Materials and Methods

The SWITCH^®^ intervention is an evidence-based implementation framework with demonstrated feasibility in school settings [13]. The primary goal of SWITCH^®^ is to increase the capacity for school leaders to implement systems and practice changes with involvement across multiple roles of school personnel, using a range of school resources, ultimately improving children’s weight-related health-promoting behaviors. All data were collected in spring of 2017 with schools enrolled in the SWITCH^®^ intervention. Approval was obtained from the Institutional Review Board (#14-651) at Iowa State University to conduct the project.

The SWITCH^®^ training and implementation framework focuses on providing schools with materials and resources to deliver wellness programming with some latitude in implementation [13]. Implementation was facilitated with the use of a customized, web-based platform that allowed students to complete online self-monitoring of their PA and SB and self-evaluate progress relative to SWITCH^®^ targets (i.e., at least 60 min of activity a day and less than two hours of SB per day). Emphasis in the present paper is on the impact of self-monitoring, which was described in SWITCH^®^ as “tracking”. Teachers also had access to curricular resources and posters to complement programming, but they were given autonomy in how they chose to use program materials.

The self-regulation process also was supported by classroom tracking participation goals and rewards called trinkets, which were provided to each classroom teacher as a means to encourage self-regulation, used at the teacher’s discretion. The standardized SWITCH^®^ training process ensures that the approach can be systematically evaluated, while the flexible implementation enables the programming to be tailored to fit local needs, interests, and capacity.

### 2.1. Sample and Measures

A convenience sample of eight rural elementary schools participated in the 2017 SWITCH^®^ feasibility study. Schools varied in size (range of total enrollment: 157 to 526 students), and socio-economic status (range of free and reduced priced lunch eligibility status: 8.5–59.4%). Consistent with the demographics in the state, however, there was limited diversity with regard to race or ethnicity (predominantly White/Caucasians; range of school-level racial/ethnic minority sample: 3.3–13.3%).

**Figure 1:**
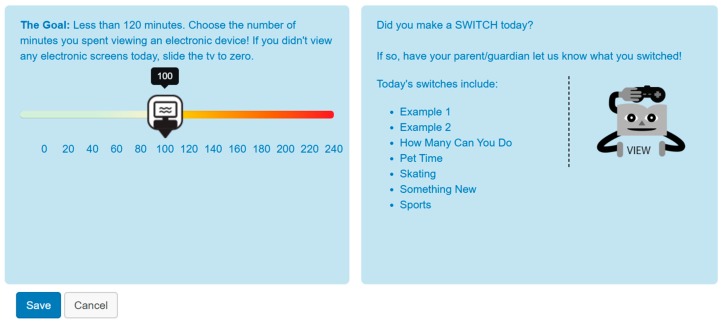
School Wellness Integration Targeting Child Health (SWITCH^®^) website self-monitoring interface.

The previous evaluation of the 2017 SWITCH^®^ iteration captured the school- and classroom-level implementation [13], so student-level engagement and outcomes were the focus for the current study. Specifically, we evaluated the relationship between self-regulation through an online platform on youth behavioral outcomes. Through the SWITCH^®^ training process, school personnel are guided through the SWITCH^®^ website and are responsible for creating classes and uploading students, who are then given login information which allows them to access the website. When students logged in, they were taken to a tracking page where they could enter behavior data daily and/or weekly, depending on their preference (see Figure 1).

Each week, students utilized the self-regulation platform for a different behavior, with the focus changing between PA, screen time, and nutrition components each week to address the targeted goals of switching “do”, “view” and “chew” behaviors (i.e., >60 min of PA, <2 h sedentary screen time, >5 fruits and vegetables). Students had opportunities to track behaviors relative to the SWITCH^®^ goals for 11 weeks in the project, but the 12th week was used to facilitate student completion of post-test measures. Once students monitored behaviors, they were provided with instantaneous feedback on whether they met daily goals (described above); such feedback manifested as green (met) or orange/red (not met). Following this, students were directed to their dashboard which shows the week at-a-glance. This platform enabled students to view how many days they had tracked and whether they met behavior-related goals for that day/week, providing immediate feedback through the website. Classrooms teachers were also able to view class-level self-monitoring and were encouraged to use trinkets as an incentive for self-regulation behaviors. Self-monitoring level was calculated using the number of weeks that students entered data into the web-based platform, divided by the number of weeks of participation in the intervention.

Physical activity outcome data were obtained using the Youth Activity Profile (YAP), a validated seven-day recall instrument developed to assess children’s PA and SB at the group level [21]. The YAP provides estimates of PA at school, during out-of-school time, and on weekends, as well as an overall estimate of SB (out of school) based on reported volitional screen time applications. Students completed the YAP assessment at pre-test (week 0) and post-test (week 12) in classroom and/or media center settings with teacher supervision. For most questions, students report their behaviors from the last seven days (e.g., “How many days did you walk to school this week?”) but other questions ask students to report their behavior during a “normal” week or day (e.g., “How active are you during recess?”). The overall YAP assessment includes 15 items with five items capturing PA in school (transportation to and from school as well as activity during physical education, lunch, and recess), five items capturing out-of-school activity (activity before school, after-school, in the evening, and on Saturday and Sunday) and five items capturing SB (watching TV, playing videogames, using the computer, using a cell phone, and general screen usage). Students were instructed to select one option from a drop-down list that best describes their behavior. An additional set of five items capturing nutrition behaviors are included in the SWITCH^®^ project, but these items have not been calibrated or formally validated. Students receive individualized feedback based on YAP raw scores but the data are processed using validated algorithms [21] to estimate time spent in moderate-to-vigorous PA (MVPA) and time spent in sedentary screen time behavior.

### 2.2. Statistical Analyses

The primary analyses focused on whether regular use of the web-based self-regulation system would positively affect youth behavior. For this reason, cases with missing data either at pre- or post-SWITCH^®^ YAP assessments were excluded. The final student sample included 513 youth (boys: *n* = 251; girls: *n* = 262) in grade 4 (*n* = 205) and grade 5 (*n* = 308) from the eight elementary schools. Only participants with complete data (pre- and post-YAP assessments) were retained in the final sample. To facilitate interpretation, we used median splits to categorize students into high or low self-monitoring categories. Although it is common to use continuous variables in linear models, the use of median splits provides a more appropriate test of the research question since self-monitoring cannot be assumed to be linearly related to behavior change [22].

All analyses were run using Proc Mixed of the Statistical Analysis System (SAS; version 9.4). The school, classroom, and student were random effects, and time was modeled as school-by-time and classroom-by-time random effects, since all schools and classrooms were exposed to intervention and seasonal weather changes over time [23]. We hypothesized that the high self-monitoring group would have better gains in PA and larger reductions in sedentary screen time behavior compared to those in the low self-monitoring group. Thus, the primary analyses focused on examining two- and three-way interactions among gender (male, female), monitoring rates (i.e., high versus low), and time (pre-winter, post-spring) on PA and sedentary screen time behavior. Five total outcomes were evaluated, including PA, PA in school, PA out of school, PA on weekends, and sedentary screen time behavior. Alpha was set at 0.05 for all analyses.

## 3. Results

The primary predictor variable of interest in the study was the rate of self-monitoring on the web-based system. The average rate of self-monitoring varied widely across schools (range: 3.6% to 89.5%) and across weeks of implementation (range: 41% to 64%) with a mean rate of 46.6 ± 33.9%. Detailed analyses of self-monitoring were provided in the implementation outcomes study [13], so the focus here is on the relationship between self-monitoring and student-level behavioral outcomes. The median rate of self-monitoring over time was 57.9%. Based on this split, students in the high self-monitoring group had monitoring rates of 88.5 ± 10.18% compared to 18.8 ± 25.71% for the low self-monitoring group. Girls were slightly more likely to be in the higher self-monitoring group than boys (55% vs 45%). Table 1 provides descriptive statistics for the pre–post changes in the key indicator variables, stratified by both monitoring status and by gender. The significance of individual changes over time are noted for each sub-group, but the main statistical comparisons of interest were the relative difference between outcomes for the two self-monitoring groups and whether the relationships varied by gender.

**Figure 2:**
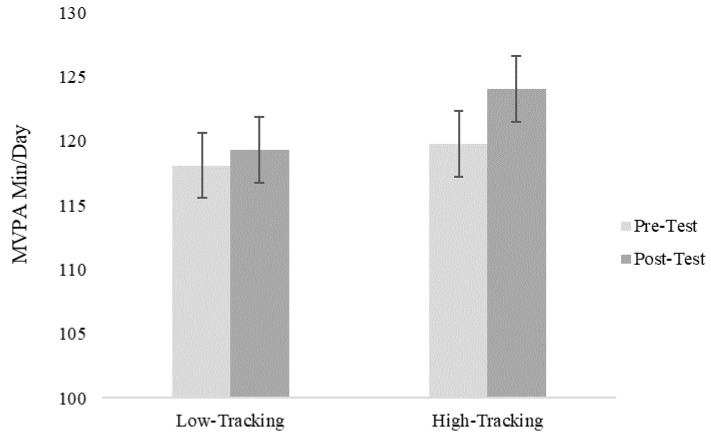
Mixed linear results for daily moderate-to-vigorous physical activity (MVPA).

For the main outcome of PA (average daily MVPA), the self-monitoring*time interaction effect was significant (F(1, 477) = 5.55, *p* = 0.02). As shown in Figure 2, the students categorized into the high self-monitoring group had larger gains in minutes of MVPA than students in the low self-monitoring group. The three-way interaction (gender*self-monitoring*time) was not significant, suggesting that the effects of self-monitoring were similar for boys and girls (F(1, 476) = 1.17, *p* = 0.27). However, it is noteworthy that both boys and girls in the high self-monitoring group had significant changes in PA (*p* < 0.01) while these changes were not significant for the low self-monitoring group.

Separate models were run to examine effects for the three sub-categories of PA captured with the YAP (i.e., in-school activity, weekday out-of-school activity and weekend activity). A significant self-monitoring*time interaction was observed for activity in school (F(1, 477) = 6.54, *p* = 0.01) with larger gains in the high self-monitoring group than the low self-monitoring group. Similar to the main outcome, the gains were significant for boys and girls in the high self-monitoring group (*p* = 0.01), but not for the low self-monitoring group (See Table 1). The self-monitoring*time interactions for activity outside of school and on weekends were not significant (*p* = 0.21) but a main effect was observed for time (F(1, 7) = 28.87, *p* = 0.001) and self-monitoring (F(1,485)= 5.26, *p* = 0.02), suggesting that the differences in the main activity outcome were mostly contained to the school setting.

**Figure 3:**
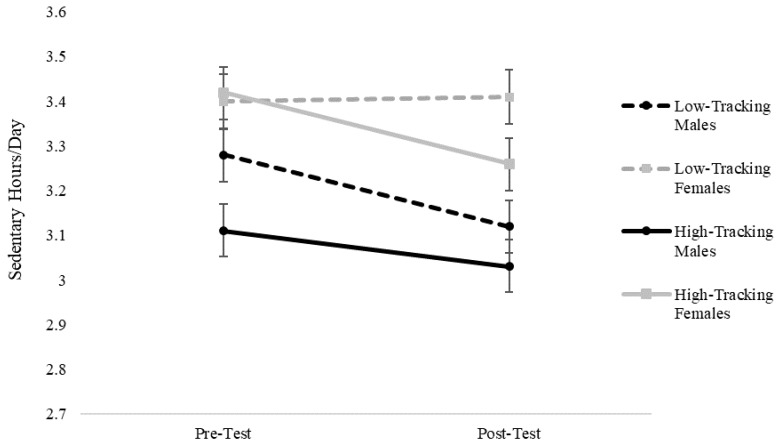
Mixed linear results for daily sedentary behavior. Note: significant gender*time*monitoring interactions (*p* < 0.01) were found that show differential relationships between boys and girls; significant main effects were found for boys (*p* = 0.03) and girls (*p* < 0.01) in the high-tracking group and boys in the low-tracking group (*p* = 0.01), but not for low-tracking girls (*p* = 0.7).

The results for sedentary screen time behavior also revealed a significant monitoring*time interaction (F(1, 477) = 4.90, *p* = 0.03). In this case, larger declines in estimates of sedentary screen time behavior were found in the high self-monitoring group compared to the low self-monitoring group. However, the three-way gender*self-monitoring*time interaction was also significant (F(1, 477) = 7.00, *p* < 0.01), demonstrating that the relationships were different between the boys and the girls. Closer examination revealed that there were significant declines in sedentary screen time behavior for boys and girls in the high self-monitoring group, as well as for boys in the low self-monitoring group, but not for girls in the low self-monitoring group (see Table 1). The nature of the three-way interaction is shown in Figure 3 to highlight the differential changes for the low self-monitoring female group.

## 4. Discussion

The purpose of this study was to test the utility of a web-based self-regulation (self-set goals, self-monitoring, described feedback fostering self-evaluation process) component for promoting PA behavior change in youth within the context of the SWITCH^®^ whole-of-school wellness initiative. Our study reinforces the premise of self-regulation as a technique to facilitate behavior change in school-based wellness programs and aligns with core tenets of SCT [18]. The results revealed that the frequency of self-regulation was associated with more positive outcomes (greater gains in PA and greater reductions in sedentary screen time behavior). Seasonality effects certainly may contribute to the overall changes observed over time since programming ran from winter to spring; nonetheless, the significant self-monitoring by time interaction supports the hypothesis that regular self-regulation facilitated by the web-based self-monitoring system may positively influence behaviors. The gains in PA were primarily due to changes in PA in school, but significant gains in PA outside of school were also evident for boys and girls in the high self-monitoring group.

To the best of our knowledge, this is the first study to demonstrate the utility of a web-based self-regulation system on changes in self-reported PA and sedentary screen time behavior in elementary school children, thus demonstrating the utility of self-regulation strategies in children. Prior research has demonstrated efficacy in fostering self-regulation among youth (elementary, middle, and high school age groups), with the goal of improving PA and nutrition behaviors [24,25,26,27,28]. For example, Lubans and colleagues facilitated self-regulation with teenage girls using pedometers to enhance their PA behavior, noting modest improvements in activity at the end of a whole-of-school intervention [27]. Previous studies have also demonstrated impacts from school-based programs on youth activity behaviors [24,29]. Scholars designing such interventions have sought to provide increased opportunities for children to be physically active, such as the “Physical Activity 4 Everyone” trial [24], whereby staff were trained to be PA leaders in their schools in children’s MVPA over the school day and outside of school. The gains in PA from the current SWITCH^®^ study were relatively modest (~4 min of MVPA per day), but this is similar to gains reported in other whole-of-school interventions targeting PA [28,29,30].

The application of SCT in the current study illustrated the potential of self-regulation as a means to foster health behavior change [17,18]. In particular, given the context in which PA and SB goal setting, self-monitoring, and feedback occurred, results demonstrated the utility of teacher-facilitated self-regulation in youth through an online platform. This may help researchers design further interventions with populations in more locations, such as rural schools, to facilitate behavior change in at-risk populations. Further, the flexible nature of the SWITCH^®^ project allows schools to implement program materials as much or as little as desired; our results reveal that self-regulation served as a behavior change strategy independent of curricular implementation at the classroom level. Accordingly, such a strategy may provide a more cost-effective solution to promoting PA behaviors in school settings where teachers become “change agents” for their students through facilitating self-regulation practices. Further research with larger, more representative samples is warranted to understand the application of self-regulation skills as a behavior change mechanism, grounded in SCT.

In addition to increases in MVPA, decreases in sedentary screen time behavior were also observed among students in the high self-monitoring group. As an outcome measure, sedentary screen time behavior is often overlooked in whole-of-school interventions. This lack of inclusion is concerning, given the linkages between SB and health risks such as obesity and type 2 diabetes [31,32] and the notion that sitting is an important risk factor for chronic disease, distinct from MVPA [33,34]. Moreover, we must acknowledge that high levels of PA do not necessarily displace sedentary time [35,36]. Thus, targeting both behaviors in school wellness programming is an important consideration.

There are several noteworthy features of the findings reported here for the SWITCH^®^ programming. One novel contribution of our study is that the design isolated the interplay between online self-regulation and children’s behavior change within the context of a broader intervention. The interaction terms provide support for the value of self-regulation as a behavior change strategy in school-based programming through the lens of SCT [17,19]. The fact that these improvements were achieved with a simple web-based self-regulation tool is also important, since it supports the inclusion of more cost-effective, web-based self-regulation platforms for scaled up whole-of-school programming. Although applications with individualized technology (e.g., pedometers, accelerometers, heart rate monitors) may have utility for behavior change, the cost would likely be prohibitive for broader dissemination in schools. A final distinction with this project is that the outcomes were achieved with the implementation of a comprehensive school-based wellness framework. Schools were provided with training on the use of the web-based platform to facilitate overall changes in school wellness, but they had autonomy for how to implement this strategy. The details on the school-level implementation were published in a separate article [13], but it is encouraging that a distributed training model can be deployed to initiate use of the online system and overall implementation of SWITCH^®^.

Although promising findings have emerged from this study with regard to the potential utility of web-based self-regulation, we must acknowledge important limitations. First, these data were gathered from predominantly Caucasian students in semi-rural and rural schools in the Midwestern United States. Therefore, findings may not be representative of schools or students in suburban and urban settings, and those of different racial and ethnic backgrounds. Second, although the YAP was calibrated against objective measures of PA with a large representative sample, there are inherent challenges associated with assessing PA and SB in youth [32]. The calibration procedures used in the YAP enable it to provide estimates of PA and SB that are statistically equivalent to estimates from accelerometer methods [21]; however, there are still limitations with all assessment methods. This is the first study to report pre-post changes with the YAP in an intervention application, so additional research with this tool is needed to evaluate the changes in individual items and the relative sensitivity to change for use in school-based interventions. Third, we did not gather data on students’ perceptions to self-monitoring; future iterations of this study will examine their attitudes and build such data into analyses. Finally, the present analyses focus on the individual outcomes associated with self-regulation rather than a system-level evaluation. We have statistically controlled for clustering at the class- and school-level, but the influence of self-regulation may be moderated by factors at the school- or class-level that are more related to implementation. Future research with larger samples will enable us to examine the contributions of school-level and class-level implementation on youth engagement as well as on the outcomes on behavior.

## 5. Conclusions

The results of the present study provide evidence for the utility of the SWITCH^®^ web-based tool and the use of self-regulation strategies for behavior change in the school setting. Youth with higher rates of self-monitoring had larger gains in PA and significant reductions in SB. The SWITCH^®^ intervention model provides a promising whole-of-school approach to school wellness, and practicing self-regulation appears to be an important element within SWITCH^®^ to promote changes in student lifestyle behaviors. Future studies of SWITCH^®^ will evaluate the dissemination and implementation of this innovative framework as it goes to scale and reaches an extensive and diverse collection of schools.

## Figures and Tables

**Table 1:** Least square mean estimates by tracking, gender and time.

Title	Low-Tracking (*n* = 237)					High-Tracking (*n* = 276)				
	Baseline Mean	Post-Intervention Mean	Adjusted Change Mean (SE)	*p* Value	95% CI	Baseline Mean	Post-Intervention Mean	Adjusted Change Mean (SE)	*p* Value	95% CI
**Male**										
MVPA/day (Min)	131.7	132.4	−0.7 (1.4)	0.6	(−3.3, 2.0)	132.1	137.1	−5.0 (1.4)	<0.01	(−7.8, −2.2)
MVPA/min/in-school day	56.3	56.5	−0.2 (1.2)	0.9	(−2.6, 2.2)	54.8	58.2	−3.3 (1.2)	<0.01	(−5.8, −0.9)
MVPA/min/out-of-school day	77.6	78.2	−0.6 (1.1)	0.6	(−2.8, 1.6)	78.2	82.1	−3.9 (1.1)	<0.01	(−6.1, −1.6)
MVPA/min/weekend day	126	127.4	−1.4 (1.6)	0.4	(−4.6, 1.7)	128.3	127.7	0.4 (1.6)	0.8	(−2.8, 3.7)
Sedentary hours/day	3.3	3.1	0.1 (0.04)	0.01	(0.02, 0.2)	3.1	3	0.1 (0.04)	0.03	(0.005, 0.15)
**Female**										
MVPA/min/day	104.5	107.2	−2.7(1.5)	0.1	(−5.6, 0.1)	106.6	111.1	−4.5 (1.3)	<0.01	(−7.1, −2.0)
MVPA/min/in-school day	45.8	45.7	0.1 (1.3)	0.9	(−2.4, 2.6)	45.8	48.1	−2.3 (1.1)	0.05	(−4.5, −0.04)
MVPA/min/out-of-school day	63.8	67.8	−4 (1.2)	<0.01	(−6.4, −1.6)	66.3	69.8	−3.6 (1.0)	<0.01	(−5.6, −1.6)
MVPA/min/weekend day	92.1	92.5	−0.4 (1.7)	0.8	(−3.9, 3.0)	91	92.2	−1.3 (1.5)	0.4	(−4.2, 1.6)
Sedentary hours/day	3.4	3.4	−0.01 (0.04)	0.7	(−0.1, 0.1)	3.4	3.3	0.2 (0.03)	<0.01	(0.1, 0.22)

Note: Mixed model with school, classroom, student and school-by-time and classroom-by-time random effects. LS change means and 95% confidence intervals (CIs) are the differences between the low-tracking group relative to the high-tracking group, adjusted for gender.

## References

[1] Bleich S.N., Vercammen K.A., Zatz L.Y., Frelier J.M., Ebbeling C.B., Peeters A. (2018). Interventions to prevent global childhood overweight and obesity: a systematic review. Lancet Diabetes Endocrinol..

[2] Amini M., Djazayery A., Majdzadeh R., Taghdisi M.-H., Jazayeri S. (2015). Effect of school-based interventions to control childhood obesity: a review of reviews. Int. J. Prev. Med..

[3] Sobol-Goldberg S., Rabinowitz J., Gross R. (2013). School-based obesity prevention programs: A meta-analysis of randomized controlled trials. Obesity.

[4] Cook H.D., Kohl H.W. (2013). Educating the Student Body: Taking Physical Activity and Physical Education to School.

[5] Centers for Disease Control and Prevention Comprehensive School Physical Activity Program (CSPAP). https://www.cdc.gov/healthyschools/physicalactivity/cspap.htm.

[6] Russ L.B., Webster C.A., Beets M.W., Phillips D.S. (2015). Systematic review and meta-analysis of multi-component interventions through schools to increase physical activity. J. Phys. Act. Heal..

[7] Webster C.A., Beets M., Weaver R.G., Vazou S., Russ L. (2015). Rethinking recommendations for implementing comprehensive school physical activity programs: a partnership model. Quest.

[8] Eisenmann J.C., Gentile D.A., Welk G.J., Callahan R., Strickland S., Walsh M., Walsh D.A. (2008). SWITCH: Rationale, design, and implementation of a community, school, and family-based intervention to modify behaviors related to childhood obesity. BMC Public Health.

[9] Gentile D.A., Welk G., Eisenmann J.C., Reimer R.A., Walsh D.A., Rusell D.W., Callahan R., Walsh M., Strickland S., Fritz K. (2009). Evaluation of a multiple ecological level child obesity prevention program: switch^®^ what you do, view, and chew. BMC Med..

[10] Welk G.J., Chen S., Nam Y.H., Weber T.E. (2015). A formative evaluation of the switch^®^ obesity prevention program: print versus online programming. BMC Obes..

[11] Dzewaltowski D.A., Karteroliotis K., Estabrooks P.A., Hill J., Welk G., Milliken G., Johnston J.A. (2009). Healthy youth places: A randomized controlled trial to determine the effectiveness of facilitating adult and youth leaders to promote physical activity and fruit and vegetable consumption in middle schools. Heal. Educ. Behav..

[12] Dzewaltowski D.A., Estabrooks P.A., Johnston J.A. (2002). Healthy youth places promoting nutrition and physical activity. Health Educ. Res..

[13] Chen S., Dzewaltowski D.A., Rosenkranz R.R., Lanningham-Foster L., Vazou S., Gentile D.A., Lee J.A., Braun K.J., Wolff M.M., Welk G.J. (2018). Feasibility study of the SWITCH implementation process for enhancing school wellness. BMC Public Health.

[14] Curran G.M., Bauer M., Mittman B., Pyne J.M., Stetler C. (2012). Effectiveness-implementation hybrid designs: combining elements of clinical effectiveness and implementation research to enhance public health impact. Med. Care.

[15] Baranowski T., Cerin E., Baranowski J. (2009). Steps in the design, development and formative evaluation of obesity prevention-related behavior change trials. Int. J. Behav. Nutr. Phys. Act..

[16] Webb T.L., Joseph J., Yardley L., Michie S. (2010). Using the internet to promote health behavior change: A systematic review and meta-analysis of the impact of theoretical basis, use of behavior change techniques, and mode of delivery on efficacy. J. Med. Internet Res..

[17] Bandura A. (1986). Social Foundations of Thought and Action: A Social Cognitive Theory.

[18] Bandura A. (2004). Health Promotion by Social Cognitive Means. Heal. Educ. Behav..

[19] Phillips L.A., Cohen J., Burns E., Abrams J., Renninger S. Self-Management of Chronic Illness: The Role of “Habit” vs Reflective Factors in Exercise and Medication Adherence. https://lib.dr.iastate.edu/cgi/viewcontent.cgi?article=1038&context=psychology_pubs.

[20] DeBusk R.F., Miller N.H., Superko H.R., Dennis C.A., Thomas R.J., Lew H.T., Berger W.E., Heller R.S., Rompf J., Gee D. (1994). A Case-Management System for Coronary Risk Factor Modification after Acute Myocardial Infarction. Ann. Intern. Med..

[21] Saint-Maurice P.F., Welk G.J. (2015). Validity and Calibration of the Youth Activity Profile. PLoS ONE.

[22] Iacobucci D., Posavac S.S., Kardes F.R., Schneider M.J., Popovich D.L. (2015). The Median Split: robust, refined, and revived. J. Consum. Psychol..

[23] Milliken G., Johnson D.E. (2009). Analysis of Messy Data Volume 1: Designed Experiments.

[24] Sutherland R., Campbell E., Lubans D., Morgan P., Nathan N., Okely A., Gillham K., Davies L., Wiggers J. (2017). ‘Physical Activity 4 Everyone’ Cluster RCT: 24-month physical activity outcomes of a school-based physical activity intervention targeting adolescents. Overall and school day physical activity outcomes. J. Sci. Med. Sport.

[25] Lubans D.R., Morgan P.J., Tudor-Locke C. (2009). A systematic review of studies using pedometers to promote physical activity among youth. Prev. Med..

[26] Marcoux M.F., Sallis J.F., McKenzie T.L., Marshall S., Armstrong C.A., Goggin K.J. (1999). Process evaluation of a physical activity self-management program for children: SPARK. Psychol. Heal..

[27] Lubans D.R., Morgan P.J., Dewar D., Collins C.E., Plotnikoff R.C., Okely A.D., Batterham M.J., Finn T., Callister R. (2010). The nutrition and enjoyable activity for teen girls (NEAT Girls) randomized controlled trial for adolescent girls from disadvantaged secondary schools: Rationale, study protocol, and baseline results. BMC Public Health.

[28] Burns R.D., Brusseau T.A., Hannon J.C. (2017). Effect of comprehensive school physical activity programming on cardio-metabolic health markers in children from low-income schools. J. Phys. Act. Heal..

[29] Telford R.M., Olive L.S., Cochrane T., Davey R., Telford R.D. (2016). Outcomes of a four-year specialist-taught physical education program on physical activity: A cluster randomized controlled trial, the look study. Int. J. Behav. Nutr. Phys. Act..

[30] Centeio E.E., Somers C., McCaughtry N., Shen B., Gutuskey L., Martin J.J., Garn A.C., Kulik N.L. (2014). Physical activity change through comprehensive school physical activity programs in urban elementary schools. J. Teach. Phys. Educ..

[31] Kriska A., Delahanty L., Edelstein S., Amodei N., Chadwick J., Copeland K., Galvin B., Haymond M., Kelsey M., Lassiter C. (2013). Sedentary behavior and physical activity in youth with recent onset of type 2 diabetes. Pediatrics.

[32] Muthuri S.K., Wachira L.-J.M., Onywera V.O., Tremblay M.S. (2015). Direct and self-reported measures of physical activity and sedentary behaviours by weight status in school-aged children: Results from ISCOLE-Kenya. Ann. Hum. Biol..

[33] Mayo Clinic (2014). Is sitting the new smoking? New science, old habit. Mayo Clin. Health Lett..

[34] Resnick H.E. (2012). Joint associations of physical activity and sedentary behavior in relation to cardiometabolic risk factors in children. (Highlights from the latest in diabetes research). Diabetes.

[35] Ekelund U., Steene-Johannessen J., Fagerland M.W., Brown W.J., Owen N., Powell K.E., Bauman A., Lee I.-M. (2016). Does physical activity attenuate, or even eliminate, the detrimental association of sitting time with mortality? A harmonised meta-analysis of data from more than 1 million men and women. Lancet.

[36] Bailey D., Fairclough S., Savory L., Denton S., Pang D., Deane C., Kerr C. (2012). Accelerometry-assessed sedentary behaviour and physical activity levels during the segmented school day in 10–14-year-old children: The HAPPY study. Eur. J. Pediatr..

